# Care seeking behaviour for childhood illness- a questionnaire survey in western Nepal

**DOI:** 10.1186/1472-698X-6-7

**Published:** 2006-05-23

**Authors:** Chandrashekhar T Sreeramareddy, Ravi P Shankar, Binu V Sreekumaran, Sonu H Subba, Hari S Joshi, Uma Ramachandran

**Affiliations:** 1 Department of Community Medicine, Manipal College of Medical Sciences, P.O. Box: 155,"Deep Heights", Pokhara, Nepal; 2 Department of Pharmacology, Manipal College of Medical Sciences, P.O. Box: 155,"Deep Heights", Pokhara, Nepal; 3 Department of Pediatrics, Manipal College of Medical Sciences, P.O. Box: 155,"Deep Heights", Pokhara, Nepal

## Abstract

**Background:**

The World Health Organization estimates that seeking prompt and appropriate care could reduce child deaths due to acute respiratory infections by 20%. The purpose of our study was to assess care seeking behaviour of the mothers during childhood illness and to determine the predictors of mother's care seeking behaviour.

**Methods:**

A cross-sectional survey was conducted in the immunization clinics of Pokhara city, Kaski district, western Nepal. A trained health worker interviewed the mothers of children suffering from illness during the preceding 15 days.

**Results:**

A total of 292 mothers were interviewed. Pharmacies (46.2%) were the most common facilities where care was sought followed by allopathic medical practitioners (26.4%). No care was sought for 8 (2.7%) children and 26 (8.9%) children received traditional/home remedies. 'Appropriate', 'prompt' and 'appropriate and prompt' care was sought by 77 (26.4%), 166 (56.8%) and 33 (11.3%) mothers respectively. The mothers were aware of fever (51%), child becoming sicker (45.2%) and drinking poorly (42.5%) as the danger signs of childhood illness. By multiple logistic regression analysis total family income, number of symptoms, mothers' education and perceived severity of illness were the predictors of care seeking behaviour.

**Conclusion:**

The results of the present study show that the mothers were more likely to seek care when they perceived the illness as 'serious'. Poor maternal knowledge of danger signs of childhood illness warrants the need for a complementary introduction of community-based Integrated Management of Childhood Illness programmes to improve family's care seeking behaviour and their ability to recognize danger signs of childhood illness. Socioeconomic development of the urban poor may overcome their financial constraints to seek 'appropriate' and 'prompt' care during the childhood illness.

## Background

Nepal is a Himalayan kingdom located between China and India. Infant mortality rate in Nepal was 64 per 1000 live births in 2001 [1]. Among childhood deaths in developing countries around 27% result from Acute Respiratory Infections (ARI) and another 23% from diarrhoea [2]. Acute diarrhoeal diseases (ADD) and ARI are most important causes of morbidity and mortality among the children in Nepal as well [1]. Various studies from developing countries have reported that delay in seeking appropriate care and not seeking any care contributes to the large number of child deaths [3-6]. Existing interventions could prevent many deaths among children if they are presented for appropriate and timely care [7]. Improving families' care seeking behaviour could contribute significantlyto reducing child mortality in developing countries. The World Health Organization estimates that seeking prompt and appropriate care could reduce child deaths due to acute respiratory infections by 20% [8]. The integrated management of childhood illness (IMCI) strategy, besides improving providers' skills in managing childhood illness also aims to improve families' care seeking behaviour. The health workers are trained to teach the mothers about danger signs and counsel them about the need to seek care promptly if these signs occur [9].

In recent years, epidemiologists and social scientists have devoted increasing attention to studying health-seeking behaviour associated with the two leading causes of child mortality, namely ADD and ARI. Health interview surveys appear to offer the best vehicle for analyzing care seeking behaviour on a representative sample of children. These surveys typically involve a single cross-sectional interviews, based on a random sample of a defined population, in which mothers are asked to report about the illnesses experienced and health services or treatment used within a specified time period prior to interview. Such health interview surveys conducted in different countries report varying results about the determinants of care seeking behaviour during childhood illness. D' Souza reports that some illnesses are categorized as 'not for-hospital'. Additionally, past experience with similar illnesses can motivate mothers to play a 'waiting game' to see if the illness subsides on its own, particularly in situations where the cost of care is an inhibitory factor [10]. Some studies have reported that care seeking behaviour is predicted by household size, age and education of the mother. Lack of access to health care due to high cost is perhaps the most common deterrent to optimal health care seeking in both rural and urban communities [11-13]. Some studies have shown that perceived illness severity, maternal recognition of certain signs and symptoms of childhood illness were critical factors determining health care-seeking behaviour [6,10,14,15]. However, Hill et al argue that health beliefs are important barriers to care seeking in addition to the maternal ability to recognize symptoms [16]. Information on the health seeking behaviour helps the policy makers set strategies to decrease the mortality due to common childhood illnesses. But to the best of our knowledge no such studies have been reported from Nepal. The purpose of our study was to assess care seeking behaviour of the mothers during childhood illness and to note the effect of sociodemographic, economic and disease-related variables, on mother's care seeking behaviour during childhood illness. We also proposed to assess the reasons for preferred care seeking behaviour and mothers' knowledge about danger signs of common childhood illnesses.

## Methods

**Figure 1:**
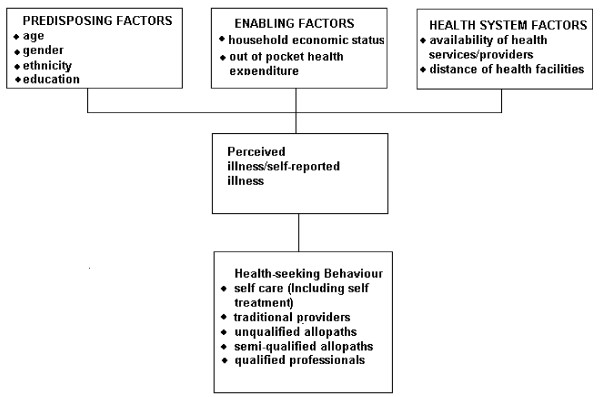
Conceptual framework for care seeking behaviour for childhood illness (Modified from Anderson) [17]:

Information about family's care seeking behaviour for common childhood illnesses is required to design appropriate child survival strategies in the areas where infant mortality rate is high. Important points to be considered in studying care seeking behaviour are as follows: (a) Proportion of families seeking care outside home and sources of care sought. (b) Appropriateness and promptness of seeking care for different illnesses; (c) The factors affecting the family's decision to seek care i.e. predisposing factors, enabling factors, health system factors and the need; (d) Finally the health beliefs about the childhood illness which influence the family's decision to seek care. See Flow Chart, Figure 1.

### Study setting and participants

Kaski district is one of the 14 districts in western development region of Nepal. The district has a land area of 2017 square kilometers and population of 380527. Kaski district has 43 village development committees and Pokhara sub metropolitan city whose population is 156312 [18]. Pokhara is administratively divided into 18 municipal wards. In each of these wards monthly immunization clinics are conducted at the child care centres. The clinics are a result of collaboration between Pokhara Municipal Corporation, United Nations International Children's Emergency Fund (UNICEF) and Manipal College of Medical Sciences (MCOMS) each providing manpower, vaccines, drugs and technical input in the form of qualified medical doctors respectively. The research ethics committee of MCOMS approved this study. A cross-sectional study was carried out in these immunization clinics of Pokhara sub metropolitan city. Since primary immunization in Nepal is completed by one year, majority of the children attending these clinics were infants. A few children who miss measles vaccine between 9 and 12 months are older than a year when they attend the Immunisation clinic. We included all the children who were brought to the immunization clinics during the months of June-July 2005. A semi-structured questionnaire was developed for the purpose of this study and was pretested among 25 mothers attending the immunization clinics. In the pre-testing phase it was evident that the information obtained from informants other than mothers was not reliable. Therefore only mothers who are care takers of the children were included for the study. After pre-testing the necessary modifications were made in the questionnaire. The questionnaire contained information about sociodemographic characteristics of the family, symptoms and duration of the illness. The mothers were also asked about the reasons for preferred type of care sought during the child's illness. The reasons for preferred type of care sought for the illness was recorded verbatim in the questionnaire. In addition mothers' were also assessed for their knowledge about the danger signs of childhood illness. The questions on the knowledge about dangers signs was adopted from the questionnaire of UNICEF multi-indicator cluster survey. [19] A health worker was trained to administer the questionnaire. The health worker was stationed in the registration counter of the clinic and enquired from the mothers about the illness suffered by the child if any during the preceding 15 days. The mothers who reported symptoms of illness in their children were informed about the study and invited to participate in the interview. After obtaining informed consent the health worker carried out the interview and recorded the necessary information in the questionnaire.

The outcome variables were classified as follows: 'Appropriate care', 'Appropriate and prompt care' and 'prompt care' based on the type of facility and qualification of the practitioner from whom the care was sought and also time interval elapsed from the recognition of illness until the care was sought/given.

The outcome variables were defined as follows:

#### Appropriate care

Care sought from qualified medical professionals in government health facilities and private hospitals/clinics.

#### Inappropriate care

Other types of care such as purchasing medicines from pharmacy, home remedies, visiting pharmacies, temples and traditional healers was defined as inappropriate care.

#### Prompt care

Any type of care that was sought/given within 24 hours from the recognition of the illness.

### Data analysis

The data was coded and analyzed using the SPSS package (SPSS Inc., Chicago, IL, USA). The results were expressed as rates and proportions. The outcomes variables were compared with sociodemographic characteristics of the family. Logistic regression analysis was carried out to identify the factors influencing care seeking behaviour with 'appropriate care' and 'prompt care' as dichotomized dependent variables and independent variables as both categorical and continuous variables. The predictors of care seeking behaviour were estimated by the calculation of odds ratios (OR) and 95% confidence intervals (CIs) and a P value of less than 0.05 was considered as significant.

## Results

Of the 1652 children who attended the immunization clinics during the study period, 292 (17.7%) children had one or more symptoms during the preceding 15 days. The mean age of the children was 6.3 months and the mean age of the mothers interviewed was 23.7 years. The mean number of symptoms reported was 1.7 and the most common symptoms reported were fever among 186 (36.7%) children, cough among 143 (28.3%) children, running nose among 74 (14.6%) children and diarrhoea among 72 (14.2%) children. Out of the 292 children who had one or more symptoms, 258 (88.4%) received some kind of care outside the home. No care was sought for 8 (2.7%) children whereas 26 (8.9%) children received traditional/home remedies. Pharmacy was the most common facility where care was sought in 135 (46.2%) episodes of illness and care was sought from qualified allopathic medical practitioners in 77 (26.4%) episodes (Table 1).

**Table 1:** Care seeking pattern for childhood illness

**Type of care sought**	**No of children**	**%**
Was taken to pharmacist for medical care	135	46.2
Was taken to medical doctor	77	26.4
Purchased medicines from a pharmacy	44	15.1
Home treatment with traditional remedies	26	8.9
Waited for illness to subside/no action taken	8	2.7
Was taken to temple/traditional healer	2	0.6
Total	292	100

Seventy seven (26.4%) out of the 292 mothers interviewed sought 'appropriate care' during the child's illness whereas 166 (56.8%) mothers sought 'prompt care' and only 33 (11.3%) mothers sought 'appropriate and prompt care'. The sociodemographic characteristics of the mothers according to the outcome variables are shown in Table 2.

**Table 2:** Sociodemographic characteristics of the mothers according to outcome variables

	**Appropriate care**		**Appropriate and prompt care**		**Prompt Care**	
	
	Yes (n = 77)	No (n = 215)	Yes (n = 33)	No (n = 259)	Yes (n = 166)	No (n = 126)
**Mother's age **Mean (SD)	23.88(3.95)	23.86(3.91)	22.72(3.51)	23.86(3.96)	23.46(3.62)	24.09(4.28)

**Religion**						

Hindu	66 (85.7)	192(89.3)	30(90.9)	228(88)	151(91)	107(84.9)
Buddhist & others	11(14.3)	23(10.7)	3(9.1)	31(12)	15(9)	19(15.1)

**Caste**						

Brahmin	18(23.4)	42(19.5)	6(18.2)	54(20.9)	34(20.5)	26(20.6)
Vaishya	12(15.6)	48(22.3)	4(12.1)	56(21.6)	31(18.7)	29(23)
Shudra	29(37.6)	70(32.6)	15(45.5)	84(32.4)	62(11.3)	37(29.4)
Chhetri	18(23.4)	55(25.6)	8(24.2)	65(25.1)	39(23.5)	34(27)

**Total family income per month (Nepalese rupees)**						

≤ 5000	15(19.5)	83(38.6)	5(15.1)	93(35.9)	51(30.7)	47(37.3)
5000–10,000	38(49.4)	84(39.1)	12(36.4)	110(42.5)	61(36.8)	61(48.4)
>10,000	24(31.1)	48(22.3)	16(48.5)	56(21.6)	54(32.5)	18(14.3)

**Mothers Education**						

No education/primary	5(56.5)	46(21.4)	2(6.1)	49(18.9)	25(15.1)	26(20.6)
High school	50(64.9)	135(62.8)	18(54.5)	167(64.5)	105(63.3)	80(63.6)
College/University	22(28.6)	34(15.8)	13(39.4)	43(16.6)	36(21.6)	20(15.8)

Figures in Parenthesis indicate percentage

1 US $ ≈ 73 Nepalese rupees

**Figure 2:**
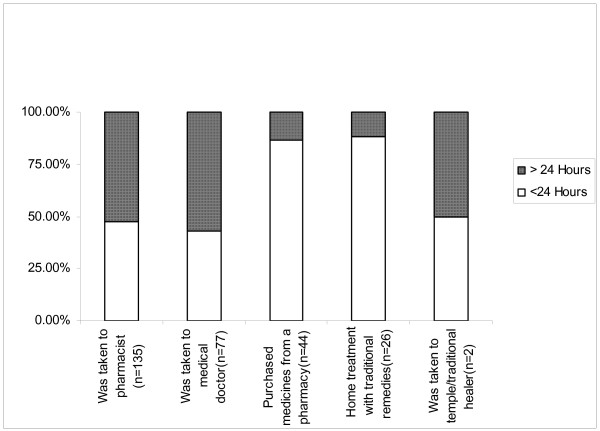
Duration after onset of illness for seeking care.

Twenty three out of the 26 (88.5%) mothers who gave home remedies and 38 out of the 44 (86.4%) mothers who purchased medicines from pharmacies gave the care within 24 hours from the recognition of illness. But only 64 out of 135 (47.4%) mothers who took the child to a pharmacist and 33 out of 77 (42.9%) mothers who took the child to medical doctor did so within 24 hours from the recognition of illness (Figure 2).

**Figure 3:**
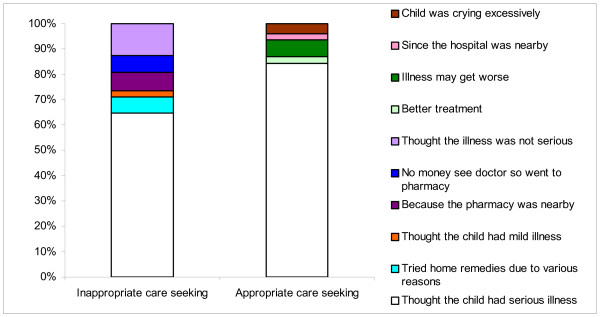
Reasons for preferred type of care sought by the mothers.

Two hundred and four out of 292 mothers (69.8%) who sought care during childhood illness reported that the reason for seeking care was 'thought child's illness was serious'. Among the 215 mothers who sought inappropriate care, 138 (64.2%) reported that they 'thought the illness was serious'; 14 (6.5%) mothers reported that they tried home remedies for various reasons. Others reasons given by the mothers for not going to a qualified medical practitioner were: 'thought the illness was not serious' (12.6%), 'pharmacy was nearby' (7.4%), 'no money to see a doctor so went to pharmacy' (6.5%). Sixty-five out of 77(84.4%) mothers who sought care from allopathic practitioners at various health facilities reported that they thought the child's illness to be serious (Figure 3).

Predictors of care seeking behaviour determined by logistic regression analysis are presented in Table 3. Maternal education, number of symptoms and perceived severity of illness were the predictors of 'appropriate care'. Family income and number of symptoms were the predictors of 'prompt care'. The mothers gave 'appropriate care' significantly more often when the child had more than one symptom, if the mother was more educated and when the illness was perceived as 'serious'. The mothers sought 'prompt care' more often when the total family income was more than 10,000 Nepali rupees per month (1 US$≈73 Nepalese rupees) and when the child had more than one symptom.

**Table 3:** Predictors of care seeking behaviour ('Appropriate care' and 'Prompt care') by logistic regression analysis

Predictor Variable	**Appropriate care**			**Prompt care**	
	
	Number/Mean(SD)	OR (95% CI)	P Value	OR (95% CI)	P Value
**Child age (months)**					

	6.25 (2.96)	0.97 (0.89 1.07)	0.66	1.07 (0.98 1.16)	0.105

**Child's gender**					

Male	157	1		1	
Female	135	1.09 (0.62 1.91)	0.76	0.9 (0.54 1.49)	0.68

**Birth order**					

	1.83 (0.95)	1.29 (0.81 2.08)	0.27	0.82 (0.58 1.15)	0.25

**Religion**					

Hindu	258	1		1	
Others	34	2.26 (0.93 5.48)	0.07	1.99 (0.87 4.57)	0.10

**Mother's age (years)**					

	23.73 (3.92)	1.0 (0.92 1.1)	0.91	1.06 (0.98 1.14)	0.14

**Mother's education**					

Nil/Primary school	51	1		1	
Upto high school	185	**7.43 (2.07 26.68)**	**0.002**	0.98 (0.43 2.01)	0.86
College/University	56	1.82 (0.89 3.71)	0.096	0.95 (0.35 2.54)	0.92

**Total Family income per month (Nepalese Rupees)**					

	12.46 (18.75)	0.99 (0.98 1.01)	0.62	**0.96 (0.94 0.99)**	**0.008**

**Number of antenatal visits**					

	3.01 (1.55)	1.02 (0.82 1.26)	0.856	0.98 (0.81 1.12)	0.89

**Place of delivery**					

Home	101	1		1	
Hospital	191	0.79 (0.39 1.59)	0.519	0.81 (0.43 1.52)	0.51

**Number of symptoms**					

1	97	1		1	
2	176	**5.43 (1.58 18.65)**	**0.007**	**2.6 (1.48 4.65)**	**0.001**
≥ 3	19	**3.38 (1.07 10.74)**	**0.038**	**5.36 (1.71 16.73)**	**0.004**

**No of living children**					

	1.85 (0.96)	1.90 (0.54,6.62)	0.309	1.50 (0.61,3.67)	0.37

**Perceived severity of illness**					

Not Severe	88	1		1	
Severe	204	**2.46 (1.21 5.03)**	**0.013**	1.6 (0.9 2.84)	0.10

1 US dollar ≈ 74 Nepalese rupees

The mothers' awareness about the danger signs of childhood illness was poor. None of the mothers were aware of all the danger signs and 10 (3.4%) mothers were not aware of any danger signs. One hundred and forty nine (51%) mothers were aware of fever, 132 (45.2%) knew about child becoming sicker and 124 (42.5%) mothers were aware about drinking poorly as the danger signs of childhood illness (Table 4).

**Table 4:** Mothers' awareness about danger signs of childhood illness

**Danger sign**	**Number of mothers aware (N = 292)**
Child develops fever	149 (51.0%)
Child becomes sicker	132 (45.2%)
Child is drinking poorly	124 (42.5%)
Child is not able to drink or breastfeed	86 (29.5%)
Child has fast breathing	83 (28.4%)
Child has difficult breathing	65 (22.3%)
Child has blood in stool	30 (10.3%)

## Discussion

With a per capita income of $240 per year, Nepal is a poor developing country in South Asia. Life expectancy at birth has increased, but at 60 years, it is still lower than the neighboring South Asian countries. Infant mortality rates are among the highest in the region. Due to high maternal mortality, life expectancy for women is lower than for men. Gender disparities are also common in terms of literacy. Only 26 percent of Nepal's women are literate, compared to 62 percent of men. There is a paucity of data on health care utilization in Nepal. The Ministry of Health estimates per capita new visits to government health facilities as 0.36 for the year 2001–02, a low level of utilization in a country which relies heavily on public provision of health care [20]. Most often, treatment for illness is sought only after home remedies have failed; women are the primary care givers at home and a large proportion of the population (42%) do not visit modern health facilities and instead seek the help of traditional healers [21]. The most recent study documenting health seeking behaviour in a hill village reports that 69% of households sought health care when an illness occurred and 26% of them visited traditional healers exclusively while only 19% first visited formal health care institutions [22]. In a previous study in western Nepal it was found that medical shop and traditional healers were common sources of medicines [23]. Traditional healers accounted for 28% of the visits to health practitioners. Respondents from low socioeconomic class from rural areas were more frequent users of complimentary medicines [24]. Self medication and non-doctor prescribing were commonly noted in a previous study [25]. The quality and effectiveness of self medication and non-doctor prescribing can be an important area of study.

In our study it was interesting to note that 46.2 % of episodes of childhood illnesses were treated by pharmacists and in 15.1% of episodes, medicines were purchased from the pharmacies without consultation. Such behaviour is not uncommon in Nepal. The International Network for Rational Use of Drugs (INRUD), Nepal has found that irrational drug use is common and is also seen in prescriptions for children less than five years of age [26]. Since many patients cannot afford to perform the tests doctors recommend, sick people or patients in places where there are no doctors consult the nearest pharmacist. In this context, we found that mothers more likely to seek appropriate care for their children when the child had more than one symptom, mothers had secondary level or higher education, and higher family income. We believe these findings are plausible. Parents are less likely to seek medical care for children with one symptom because many of these symptoms resolve on their own. When we compared our findings with those of other published studies, we found both supporting and contrasting results. Most studies have identified economic status as the most significant predictor of healthcare utilization. For example, Neumark et al identified economic status as the determining factor for the number of visits to medical facilities [27]. Our study identified total family income as a predictor of care seeking behaviour. But Pillai et al have reported from Kerala, southern India that families with a higher economic status might seek care less often, particularly for milder illnesses because the family has the resources needed to obtain care later in the illness if it does not resolve [28].

Several studies have reported a positive relationship between maternal education and care seeking behaviour which is in agreement with the present study [29,30]. Lesser education of mother has been associated with lower utilization of health services. But, Pillai et al have found a negative relationship between maternal education and care-seeking behaviour [28]. A possible explanation for such behaviour could be that better educated mothers usually have higher income so they may wait for the illness to subside spontaneously or have enough resources for treatment if the illness gets worse. However in our study we found that children having more than one symptom were more likely to receive appropriate and prompt care. A few studies have considered maternal perceived severity of illness as a factor influencing care seeking behaviour [15,28,31]. But some studies have reported that mother's perceived severity of illness is not reliable in terms of recognition and interpretation of severity of illness [16,32]. Therefore we considered both number of symptoms and mother's perception of severity of illness in the regression analysis to identify the predictors of care seeking behavior. Perception of severity of illness was a predictor for appropriate care but not prompt care. Income was a major deterrent to these urban mothers to seek prompt care.

Pokhrel et al. reported that gender of the child not only affects illness reporting but also affects the decision to choose a health care provider and also how much to spend on the sick child [33]. However we did not find any such association in our study. Pokhrel & Sauerborn have reported that in Nepal the effect of cost of healthcare on decisions about whether to utilize healthcare services for children was statistically significant and subsidy policies could improve utilization substantially [34]. Antenatal visits might be an indicator of the utilization of all types of care because some families want more medical care than others and some families have easier access to antenatal and other care because of proximity, or because of other reasons. However in our study number of antenatal visits was not a predictor of care seeking behaviour.

One of the strategies of IMCI to reduce the under-5 child mortality is education of the mother and/or caregiver on home care of the child during illness and after recovery, and on the signs of severe illness for which the child should be taken immediately to a health worker. In this context we assessed the mothers' awareness about the danger signs of childhood illness. Overall the knowledge of mothers was poor. This was despite 241 (82%) mothers having education of secondary or higher level. However some mothers mentioned that 'cough', 'pneumonia', 'jaundice' as the danger signs though they were not aware of the specific danger signs. Although mothers generally recognized the childhood illness as serious, a large proportion of them did not seek appropriate and prompt care. Mohan et al have reported that training doctors in counselling mothers can improve their appreciation of the need to seek prompt and appropriate care for serious episodes of childhood illness [35]. Community-based intensive behavioral communication strategies complementing clinic-based IMCI programmes can reinforce mothers' perception of illness severity and enhance their cues to appropriate action [16].

It is important to understand the limitations of our study. In our study we relied on self-reported answers, and these may be subject to recall and reporting bias. Our predictive model does not control for health beliefs and quality of care, which may play a role in determining health services utilization. Our categorization of the appropriate and inappropriate care was not homogenous and included a diverse array of providers. Unidentified predictors or confounders are still possible regarding attitude and perceptions of people about illness and health seeking behavior. Further research is needed to improve upon these limitations.

## Conclusion

A large proportion of mothers did not seek 'appropriate' and 'prompt' care for childhood illness and most often the care was sought from pharmacies instead of from qualified medical practitioners. The mothers tended to seek 'appropriate care' more often when they perceived the illness as 'serious'. However, a large proportion of the mothers were not aware of the danger signs of the childhood illness. Total family income, mother's education, number of symptoms and mother's perceptions about severity of illness were the predictors of the seeking behaviour. Complementary introduction of community-based IMCI programmes may improve family's care seeking behaviour and their ability to recognize danger signs of childhood illness. Socioeconomic development of the urban community can improve care seeking behaviour during the childhood illness.

## Abbreviations

IMCI: Integrated Management of Childhood Illness

ARI: Acute Respiratory tract Infection

ADD: Acute Diarrhoeal Disease

UNICEF: United Nations International Children's Emergency Fund

## Competing interests

The author(s) declare that we have no competing interests.

## Authors' contributions

CTS contributed to the design and protocol of the study, participated in the data collection, was the primary researcher did the analysis and drafted the final manuscript.

PRS contributed to the protocol, made a substantial contribution to the analysis drafting of the manuscript and corrected the initial and final drafts of the manuscript

BVS provided assistance with the analysis and the manuscript preparation.

SHS helped with the design of the study and development of questionnaire.

HSJ helped with the design, the protocol and the data collection.

UR conceived the study, set up the design, guided and participated in the analysis and revised the final manuscript.

All authors read and approved the final manuscript for submission for Publication.

## Pre-publication history

The pre-publication history for this paper can be accessed here:


